# Differentiated Embryo-Chondrocyte Expressed Gene1 and Parkinson’s Disease: New Insights and Therapeutic Perspectives

**DOI:** 10.2174/1570159X21666230502123729

**Published:** 2023-09-01

**Authors:** Chun-Yan Wang, Zheng-Jie Qiu, Ping Zhang, Xiao-Qing Tang

**Affiliations:** 1 Institute of Cardiovascular Disease, Key Laboratory for Arteriosclerology of Hunan Province, Hunan International Scientific and Technological Cooperation Base of Arteriosclerotic Disease, Hunan Province Cooperative Innovation Center for Molecular Target New Drug Study, Hengyang Medical College, University of South China, Hengyang, Hunan 421001, China;; 2 The Affiliated Nanhua Hospital, Department of Neurology, Hengyang Medical School, University of South China, Hengyang, Hunan 421001, China;; 3 Hengyang Key Laboratory of Neurodegeneration and Cognitive Impairment, Institute of Neuroscience, Hengyang Medical College, University of South China, Hengyang, Hunan 421001, China

**Keywords:** Parkinson's disease, neurodegenerative disease, central nervous system, pathogenesis, transcription factor, DEC1

## Abstract

Differentiated embryo-chondrocyte expressed gene1 (DEC1), an important transcription factor with a basic helix-loop-helix domain, is ubiquitously expressed in both human embryonic and adult tissues. DEC1 is involved in neural differentiation and neural maturation in the central nervous system (CNS). Recent studies suggest that DEC1 protects against Parkinson's disease (PD) by regulating apoptosis, oxidative stress, lipid metabolism, immune system, and glucose metabolism disorders. In this review, we summarize the recent progress on the role of DEC1 in the pathogenesis of PD and provide new insights into the prevention and treatment of PD and neurodegenerative diseases.

## 1 INTRODUCTION

Parkinson's disease (PD) is the second most common degenerative disease. As the global population is growing and ageing, PD is the fastest-growing neurological disorder, and as of 2016, 6.1 million people worldwide sufferred from this disease [1]. It is well known that PD is characterized by the death of dopaminergic neurons in the substantia nigra and the formation of the Lewy body consisting of an aggregation of the α-synuclein protein. PD is a heterogeneous disease with both rapidly and slowly progressive forms. The exact etiology of PD is still unclear and may be related to genetic, environmental, behavioral, age, aging of the nervous system, and other factors [2]. At present, there is no effective treatment for this disease. And the current treatment is symptomatic, focusing on the dopamine-based treatment of initial motor symptoms and nondopaminergic approaches to the treatment of nonmotor symptoms (For example, selective serotonin reuptake inhibitors for psychiatric symptoms and cholinesterase inhibitors for cognitive behavior), as well as rehabilitation and exercise supplement therapy [3]. Therefore, an in-depth understanding of the roles of pivotal molecules in the lesion formation of PD and the exploration of new prevention and treatment targets are of great importance to designing new promising therapeutic strategies for PD.

**Fig. (1):**
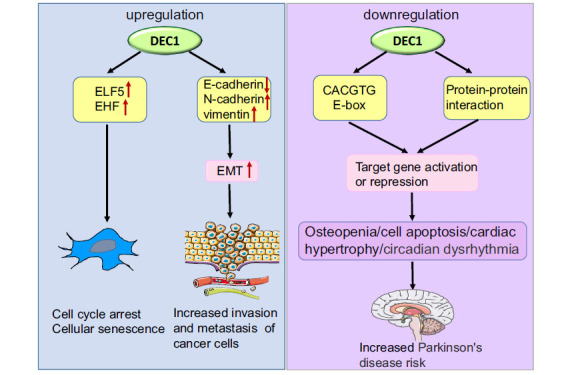
Fine-tuning of DEC1 for different disease states. Upregulation of DEC1 facilitates invasion and metastasis of cancer cells by downregulating E-cadherin, upregulating N-cadherin, vimentin, and inducing EMT. DEC1 overexpression promotes cell growth cycle arrest and senescence by promoting the expression of target genes ELF5 and EHF. DEC1 promotes target gene repression or activation by interacting with other proteins or by direct binding to (CACGTG) E-box. Downregulation of DEC1 is associated with osteopenia, cell apoptosis, cardiac hypertrophy, and circadian dysrhythmia, resulting in increased PD risk. DEC1, differentiated embryo-chondrocyte expressed gene 1; E-cadherin, epithelial cadherin; N-cadherin, neural cadherin; EMT, epithelial-mesenchymal transition; (CACGTG) E-box, the CACGTG E-box sequence in the promoter regions of various target genes.

Differentiated embryonic chondrocyte expressed gene 1 (DEC1, also known as Sharp2/Stra13/BHLHE40/ Bhlhb2), is a transcription factor containing 412 amino acids with a basic helix-loop-helix (bHLH) structural domain. The basic/helix-loop-helix (bHLH) proteins are a superfamily of transcription factors that bind as dimers to specific DNA target sites. This family of proteins is characterized by the common possession of highly conserved bipartite domains for DNA binding and protein-protein interaction. The basic region: A motif of mainly basic residues permits helix-loop-helix proteins to bind to a consensus hexanucleotide E-box (CANNTG). The HLH region: A motif of primarily hydrophobic residues referred to as the helix-loop-helix domain allows these proteins to interact and form homo- and/or heterodimers [4]. They are involved in the regulation of neurogenesis, myogenesis, cell proliferation and differentiation, cell lineage determination, sex determination, and other essential processes in organisms ranging from yeast to mammals [5]. The DEC1 protein is a member of a new subgroup of the proline bHLH protein family that diverged earlier than other proline bHLH proteins including HES, hairy, and E(spl) [6]. DEC1 was cloned from differentiated human embryonic chondrocytes in 1997 [7]. It is also named stimulated with retinoic acid 13 (STRA13) in mice [8] and split and hairy related protein 2 (SHARP2) in rats [9]. As a member of the bHLH family, DEC1 is widely expressed in various organs and tissues throughout the body and is involved in various physiological activities of the human body, including neuronal cell formation, chondrocyte differentiation [10], biological clock regulation [11], immune response [12], lipid metabolism [13], and cell proliferation and differentiation [14]. In addition, numerous studies have confirmed that DEC1 plays a key role in tumor development [15] and cell survival [16]. DEC1 was upregulated in tumor cells, inducing EMT and senescence apoptosis of tumor cells [17, 18]. Indeed, DEC1 plays different roles in different tissues and organs, so it can be “fine-tuned” in different disease states (Fig. **1**). In recent years, accumulating evidence has shown that DEC1 is closely related to inflammatory diseases of the CNS [19], such as encephalomyelitis, Parkinson's disease, glioblastoma [20], psychiatric diseases, and other neurological diseases [21].

DEC1 is a sequence-specific transcriptional inhibitor that regulates the transcription of its target genes through direct DNA binding. On the one hand, DEC1 mainly acts as a transcriptional repressor by directly binding to class B E-boxes (CACGTG) [22] or by recruiting histone deacetylases (HDACs) as co-repressors [23]. Moreover, DEC1 interacts with components of the basal transcription machinery, such as TFIIB, TBP, and TFIID, and exerts transcriptional repression [24, 25]. On the other hand, DEC1 is capable of activating surviving [26] and DeltaNp63 [27] gene expression *via* binding to the Sp1 sites. In addition, DEC1 can also activate the expression of TAp73 gene (tumor protein 73, p73 isoform containing transactivation domain) by interacting with HDAC8 [28]. These studies indicate that DEC1 functions as both transcriptional activator and repressor in regulating various genes by utilizing different responsive elements and associating with different transcriptional co-factors. Upon binding to these proteins, DEC1 regulates the biological functions of effector cells, thereby affecting the development of diseases. Thus, DEC1 may become an attractive target for the prevention and treatment of neurological diseases. This review summarized the structure and function of DEC1 and the current understanding of the pathophysiological and etiological roles of DEC1 in PD and tried to provide evidence for future research and therapeutic interventions.

## 2 STRUCTURAL CHARACTERISTICS OF DEC1

**Fig. (2):**
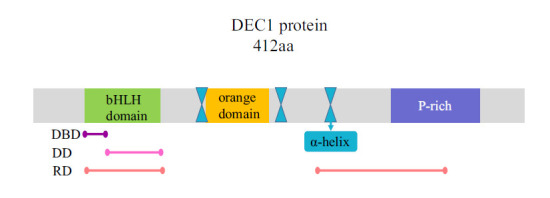
Deduced amino acid sequences of mammalian DEC1. bHLH, basic helix-loop-helix motif, responsible for the e-box binding region and inhibiting transcriptional activity; orange, orange domain; P-rich, proline-rich domain; α-helix, three α-helices; DBD, DNA-binding domain; DD, dimerization domain; RD, repression domain.

DEC1 is a novel cAMP-inducible basic helix-loop-helix (bHLH) transcriptional factor. The DEC1 gene is located on chromosome 3p25.3-p26. The human DEC1 gene is about 5.7 KB in length and contains 5 exons and 4 introns. All introns are located within the protein-coding region and among the four introns, only intron 3 is located within the bHLH domain; introns 1 and 2 are located between the N-terminal and the bHLH domain, and intron 4 is located at the C-terminal of the bHLH domain [6]. DEC1 contains 412 amino acids, which are composed of the N-terminal domain (DEC1:1-139) including the bHLH domain, and the C-terminal domain (DEC1:140-412) including the orange domain (Fig. **2**). The N-terminal domain of DEC1 is essential for the function of DEC1, especially the bHLH domain, which is an important region for inhibiting transcriptional activity, interacting with BMAL1, and binding of E-box (CACGTG) [29]. DEC1 lacks the WRPW motif responsible for the recruitment of the co-repressor Groucho in other E-class members of the bHLH family [8]. Instead, its inhibitory activity is mediated by the three α-helices located at the C-terminal and represses transcription through two distinct mechanisms. One is that DEC1 expression is transcriptionally repressed through interaction with the corepressor histone deacetylase (HDAC1) by its C-terminal a-helices. The other is that DEC1 acts as a repressor of c-Myc expression by an HDAC-independent mechanism that involves interaction with TFIIB (transcription factor IIB) [16]. DEC1 can interact with transcription coactivators P/CAF (P300/CBP-associated factor) and this interaction can rescue DEC1-mediated inhibition of target gene transactivation [30]. DEC1 can bind to BDNF (Brain-derived neurotrophic factor) gene promoter 4 to negatively regulate BDNF transcription and alter neuronal excitability, which has important implications for regulating the development of neurodegenerative diseases [31].

## 3 POST-TRANSLATIONAL MODIFICATIONS OF DEC1

**Fig. (3):**
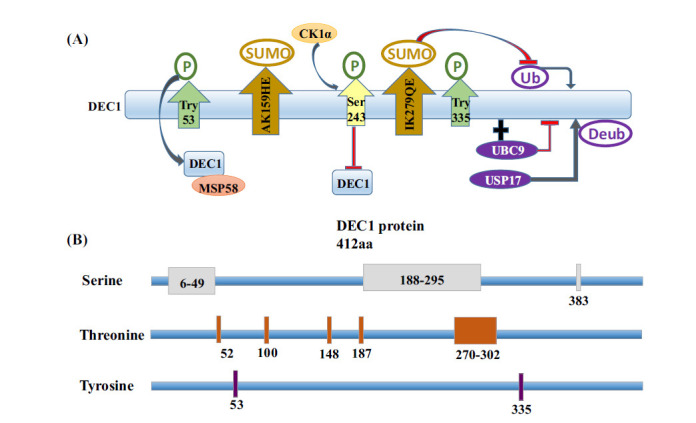
Post-translational modifications of DEC1. (**A**) Tyrosine phosphorylation sites are located at 53 and 335 amino acids of DEC1. Casein kinase (CK1α) inhibits DEC1 activity by phosphorylation of Ser 243. Phosphorylated DEC1 can increase the stable binding of DEC1 to MSP58 protein to form a transcriptional inhibitory complex. DEC1 binds to UBC9 (ubiquitin-binding enzyme) and proteolyzes DEC1. USP17 ubiquitin-specific protease 17 deubiquitinated and stabilized DEC1. Two major SUMOylation sites: AK159HE and IK279QE. SUMOylation of DEC1 enhances the stability of DEC1 by inhibiting its ubiquitination. P: phosphorylation; SUMO: SUMOylation; Ub: ubiquitination; Deub: deubiquitylation. 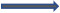: promote; 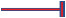: inhibit. (**B**) Localization of the phosphorylation sites in the DEC1 protein sequences. Numbers reflect amino acid residues.

DEC1 functions as both a transcriptional activator and suppressor, depending on interacting with proteins or acting cis-element [32]. However, the molecular mechanism of choice between activation and inhibition of DEC1 in the nervous system is unclear, but it may involve changes in the post-translational modification function of DEC1, thus affecting its regulation of target genes (Fig. **3A**).

### 3.1 Phosphorylation of DEC1

DEC1 has many predicted phosphorylation sites, such as most serine and threonine phosphorylation sites, located at the N-terminal 300 amino acids of DEC1, and two functional tyrosine phosphorylation sites, located at 53 and 335 amino acids of DEC1 [33] (Fig. **3B**). Casein kinase (CK1α) is involved in the degradation of DEC1 by specifically phosphorylating DEC1 Ser243. Increased phosphorylation of DEC1 has been shown to increase the stable binding of DEC1 to MSP58 protein to form a transcriptional inhibitory complex protected from protein degradation and thus exert transcriptional inhibition. And only tyrosine Tyr53 or serine/ threonine phosphorylation of the DEC1 basic domain may be responsible for the binding modulation. Additionally, tissue expression pattern analysis indicated that MSP58 is expressed strongly in rat brains and localized in the cytoplasm of neurons. And the presence of MSP58 in synaptic polysomes increases its ability to control local de novo protein synthesis, a fundamental phenomenon of synaptic development and maturation [34]. These studies suggest that the phosphorylation status of DEC1 affects its stable binding to MSP58 protein molecules, thus playing a key role in the nervous system.

### 3.2 Ubiquitin Modification of DEC1

Ubiquitination is a key post-translational modification that regulates subcellular localization, stability, and transcriptional activity of transcription factors [35]. DEC1 is targeted for proteolysis by the ubiquitin-dependent proteasome pathway through association with UBC9 (ubiquitin-conjugating enzyme) [23]. The DNA damage-induced increase in DEC1 is the result of protein stabilization through the mechanism of the USP17 ubiquitin protease. USP17 (ubiquitin-specific protease 17) is identified as a deubiquitinating enzyme that deubiquitinated and stabilized DEC1 [36]. Together, these results suggest that the regulated degradation of DEC1 is a key factor controlling the DNA damage response. Although it has been confirmed that dysfunction of the ubiquitin-proteasome system and DNA damage is associated with the development of PD [37, 38], it is unclear whether ubiquitin modification of DEC1 affects the progression of PD. Therefore, it must be emphasized that future studies will be required to precisely define the roles of ubiquitin modification of DEC1 in PD.

### 3.3 SUMOylation of DEC1

SUMOylation is an important post-translational modification that modulates the biological functions of proteins. Two major SUMOylation sites, AK^_159_^HE and IK^_279_^QE are present at the C-terminus of DEC1. The protein DEC1 can be modified by SUMO1, 2, and 3 at these two sites. SUMOylation of DEC1 enhances the stability of DEC1 by inhibiting its ubiquitination [39]. Since both SUMOylation and ubiquitin modification are lysine-targeting, the antagonistic relationship between SUMOylation and ubiquitin modification may play an important role in regulating DEC1 activity. Several lines of evidence suggest that the SUMO system exerts a protective function in hypoxia. The hyper SUMOylation of transcription factor DEC1 in hypoxia facilitates metabolic adaptations and contributes to the inhibitory effect of hypoxia on mitochondrial aerobic metabolism through the repression of genes involved in oxidative metabolism [40]. The strong increase in SUMO conjugation observed in mouse models of cerebral or cardiac ischemia, as well as in cellular models of ischemia, is mainly regarded as a tolerance mechanism against hypoxia [41-43]. In addition, it has already been reported that the capacity of hypoxia conditioning increases cellular resilience to hypoxic, ischemic, and possibly proteotoxic stress. And hypoxia conditioning is a promising potential treatment for neurological disorders, particularly neurodegenerative diseases [44]. Together, these studies indicate that SUMOylation of DEC1 regulates its transcriptional inhibitory activity and may play a neuroprotective role in neurodegenerative diseases. In conclusion, understanding the structural characteristics and post-translational modifications of DEC1 will contribute to a better understanding of its biological functions in neurological diseases.

## 4 THE CIRCADIAN RHYTHMS REGULATORY ROLE OF DEC1 IN NEUROPHYSIOLOGICAL DEVELOPMENT

DEC1 plays a significant role in neuronal differentiation, especially in the endoderm and mesoderm stages. P19 cells serve as a model system to study the *in vitro* differentiation of nerve cells. On the one side, P19 cells can differentiate into all germ layers under the induction of Retinoic acid (RA) [45], including endoderm, mesoderm, and ectoderm. DEC1 acts as a transcription inhibitor and inhibits mesoderm and endoderm differentiation. On the other side, aggregated P19 cells treated with RA can differentiate into similar neurons, glial cells, and fibroblasts. DEC1 and RA co-induce neuronal differentiation of P19 cells. At the same time, Stra13 cells differentiate into neuron-like cells when treated with RA or aggregated in the absence of RA, so DEC1 overexpression promotes neuronal differentiation of P19 cells [8].

DEC1 is thought to be a clock protein that regulates circadian rhythms by regulating clock output signals to control circadian rhythms in behavior and metabolism. The expression of *Dec1* is induced by CLOCK/BMAL1 heterodimer through the E-box of CACGTG and inhibited by the direct binding of their products to the same E-box [46]. Among the molecular mechanisms that regulate circadian rhythms, CLOCK/BMAL increases the promoter activity of DEC1, and DEC1 suppresses CLOCK/BMAL-induced *Per* transactivation through competition for E-boxes and/or protein-protein interaction with BMAL1 [47]. *Dec1* overexpression causes a phase delay in the circadian rhythm of CACGTG E-box-containing gene expression, including *Dec1*, *Dec2*, *Per1*, *Dbp*, and *Rev-erbα*. These all turned out to be clock-controlling genes in the molecular clock system. But the lack of *Dec1* advances the circadian rhythm of E-box-containing [11]. In addition, the locomotor activities of mice were affected by the disruption of the Dec1 gene. Melatonin, a major marker of circadian rhythms, could be a potential therapeutic drug in PD [48]. Interestingly, Ramelteon (melatonin receptor agonist) treatment could adjust the circadian rhythm of patients with sleep disturbance comorbid with anxiety and depression and normalize the expression levels of DEC1 [49]. These findings suggest that changes in clock gene DEC1 in circadian rhythms may potentially affect the circadian development of PD.

It is worth noting that circadian rhythms play a critical role in nervous system development and neuron differentiation. Many studies have observed that the core clock mechanism is present in neurons and astrocytes [50, 51]. Recent studies suggest that cellular circadian clocks may regulate adult neurogenesis, differentiation, and survival of newly formed neurons [52, 53]. Circadian clock genes regulate cognitive function by regulating the expression of neural-plasticity genes, such as the brain-derived neurotrophic factor (BDNF) gene and its high-affinity receptor tyrosine kinase B (TrkB) [54]. Circadian disturbances are a novel environmental risk factor for the neuropathology of PD [55]. Circadian fluctuations in PD cause abnormalities in movement, vision, blood pressure, and heart rate. For example, motor dysfunction is less severe in the morning than in the afternoon; circadian variability in visual contrast sensitivity [56]; hypertension and heart rate can be observed during light periods, while hypotension can be observed during dark periods [57]. Altogether, circadian rhythm disturbance can induce Parkinson's disease in multiple ways, as shown in Fig. (**4**). Because of this, modulation of biological circadian effects may be therapeutic for PD. However, the specific mechanism or target still needs to be further explored.

Furthermore, current evidence suggests that sleep plays a critical role in the pathophysiology of neurodegenerative diseases. Sleep disruption is a central aspect of neurodegenerative disorder prodromes [58-60]. Overnight changes in sleep architecture in PD may accelerate PD-associated cognitive decline through worsening consolidation of memory and poor maintenance of protein clearance, synaptic homeostasis, and plasticity [61]. Sleep disturbance and autonomic dysfunction are related to the pathological changes of the molecular clock in circadian rhythm in early PD, resulting from the lack of dopamine that directly affects the central components of the molecular clock [62, 63]. Striatal dopamine controls the Bmal1/Clock heterodimer expression in a receptor-dependent manner [64]. In parallel, dopamine can regulate the rhythmicity of Per2 expression [65]. In PD, elevated cortisol and reduced melatonin are associated with altered *Bmal1* expression [62], and reduced expression is correlated with disease severity [66]. These studies indicate that sleep disorders affect PD and are closely related to circadian rhythm disturbances. There is a dual necessity to explore sleep disturbances in PD: first, to enable the development of circadian-facing therapeutic treatments, and second, to study the mechanisms of interaction between sleep and disease progression. As such, sleep dysfunction in PD represents a so far untapped potential therapeutic target.

**Fig. (4):**
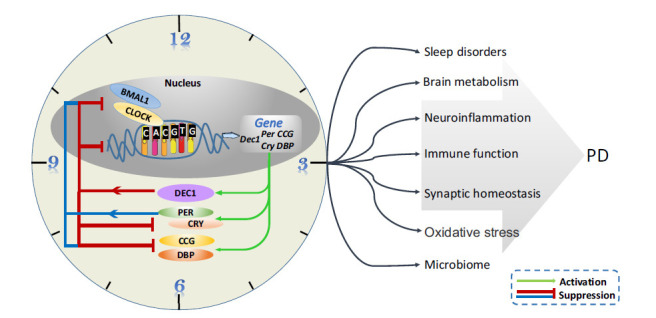
The underlying mechanisms of the circadian clock influencing PD. A heterodimer of CLOCK and BMAL1 binds to the E box sequence (CACGTG) and stimulates the expression of the clock or clock-related genes, *Dec1*, *PER*, *CRY*, some *clock-controlled genes* (*CCG*), and *DBP*. Meanwhile, DEC1 act as repressor to form the DEC loop. PER/CRY heterodimer also suppresses CLOCK/BMAL1-induced transcription of the *Per* genes to form the PER loop. The core circadian clock may contribute to neurodegeneration and PD by affecting sleep duration, inflammation, oxidative stress, synaptic function, and regulating microbial and brain metabolic responses. PER, period circadian protein homolog; CRY, cryptochrome circadian clock; CCG, clock-controlled gene; DBP, D-binding protein.

To sum up, sleep and circadian rhythm dysfunction present near ubiquitously in the clinical setting. In this area, there is growing support for the notion that not only are circadian and sleep dysfunction two consequences of PD, but both may also play a predisposing role, inducing onset and exacerbating disease progress, respectively [67]. Although there is no direct and sufficient evidence to prove the role of DEC1-mediated circadian rhythm regulation in PD, DEC1 may play an important role in nervous system development and PD by regulating other clock-controlling genes (Fig. **4**). In terms of future directions, there is a need for further detailed investigation of circadian alterations and sleep disorders in PD, utilizing modern research methodologies and tools, to elicit insight into both mechanisms of disease progression and potential therapeutic treatments.

## 5 THE ANTI-PARKINSON'S DISEASE ACTION OF DEC1

PD is a common degenerative disease of the elderly nervous system. Several lines of evidence demonstrate that DEC1 is tightly associated with PD. Serine-Threonine Protein Kinase Akt signaling plays an important role in the development of PD. Numerous studies have shown that Akt signaling activation plays a neuroprotective role by regulating several processes, including promoting neuronal survival [68], increasing GDNF expression [69], inhibiting microglia-induced neurotoxicity [70], inhibiting dopaminergic cell death [71], and regulating homeostatic dopaminergic transporters [72]. Importantly, in DEC1-deficient mice, PI3K/ Akt/GSK3β signaling pathway was significantly inhibited. At the same time, DEC1 deficient mice exhibit loss of midbrain dopaminergic neurons in the substantia nigra pars compacta (SNpc) and reduction of dopamine in the striatum [73], suggesting the role of DEC1 in PD is achieved through the regulation of the Akt signaling pathway. Furthermore, the expression of DEC1 significantly is decreased in the established subacute MPTP-induced mouse model of PD with motor impairment. And DEC1 downregulation is involved in MPP^+^-induced neurotoxicity and leads to cell death by inhibiting the PI3K/Akt/GSK3β signaling pathway [74], suggesting the neuroprotective effect of DEC1 is achieved through inhibiting the PI3K/Akt signaling pathways.

The Liver X receptor (LXRβ) plays an important role in the CNS and immune system [75-78], and its loss of function mutation is associated with PD. LXRβ deficiency in mice increases MPTP-induced dopaminergic neurotoxicity [79]. Additionally, a lot of literature has suggested that LXRβ agonists may be a new treatment for neurodegenerative diseases such as PD [80, 81]. Notably, it has been found that LXRα and LXRβ are potent enhancers for *Dec1* promoter activity [82]. Taken together, these findings suggest that the anti-PD effect of LXR agonists involves its positive regulation of DEC1 expression. Therefore, the increased expression of DEC1 plays a positive role in anti-PD. In addition, the knockdown of DEC1 reduces hyperactivity behaviors in SHR, a well-established animal model of ADHD (attention deficit hyperactivity disorder) [83]. Since bradykinesia and stiffness are the most prominent symptoms of PD, it is suggested that a deficiency of DEC1 may contribute to Parkinson's dyskinesia. Taken together, these studies suggest that DEC1 plays a protective role in the development of PD and that targeting DEC1 may be a promising strategy for treating PD.

## 6 THE POTENTIAL MECHANISMS UNDERLYING THE ANTAGONISTIC ACTION OF DEC1 IN PD

The etiology of PD is a complex and multifactorial process involving apoptosis, oxidative stress, lipid metabolism, immune system, and glucose metabolism disorders. DEC1 is shown to protect against PD by multiple mechanisms.

### 6.1 DEC1 Inhibits Apoptosis

Apoptosis is a programmed, controlled process of cellular self-destruction and a key process of cellular homeostasis that requires protein synthesis and specific cellular signals and proteins [84]. More and more evidence indicates that apoptosis plays an important role in the death of substantia nigra dopaminergic neurons in PD. Especially, the apoptosis of substantia nigra dopaminergic neurons promotes the occurrence and development of PD, and the amount of apoptosis of substantia nigra neurons increases with the increase of neurotoxicity [85-87]. It has been found that down-regulation of DEC1 is involved in inducing apoptosis both *in vivo* [88] and *in vitro* [18]. In addition, secretory clusterin (sCLU) protein, a pro-survival activity that inhibits apoptosis, is identified as a direct target gene of DEC1, and both CLU mRNA and sCLU protein can be upregulated by overexpressed DEC1 [89]. These results suggest that DEC1 inhibits apoptosis at least to a certain extent by promoting the expression of sCLU. Furthermore, Survivin is an important member of the apoptosis inhibitor (IAP) family and a direct target of DEC1 and targeting this protein protects neurons from apoptotic death [90]. DEC1 can act as a transcriptional activator to up-regulate the expression of Survivin, thereby antagonizing apoptosis [91]. Other studies have shown that the Wnt protein promotes the differentiation of midbrain dopaminergic (DAergic) neurons and maintains the survival of SN DAergic neurons by activating the Wnt/β-catenin signaling cascade [92, 93]. In an animal model of familial Parkinson's disease type 17 (PARK17) presenting with autosomal dominant inheritance and delayed Parkinson's syndrome, PARK17 mutant (D620N) VPS35 induces degeneration and death of SNpc DAergic neurons by impairing the activity of Wnt/β-catenin signaling cascade [94]. It has been also found that DEC1 knockdown significantly inhibits the Wnt/β-catenin pathway [95]. These show that DEC1 can regulate the Wnt/ β-catenin signaling pathway to maintain neuronal survival and thus resist apoptotic death. Collectively, these results suggest that DEC1 inhibits the progression of PD by acting as an anti-apoptotic agent through a variety of mechanisms.

### 6.2 DEC1 Resists Oxidative Damage

Oxidative stress reflects an imbalance between the overproduction of free radicals and the dynamic ability of biological systems to detoxify active intermediates. Free radicals produced by oxidative stress affect the structure and function of nerve cells and contribute to a range of neurodegenerative diseases, including Parkinson's disease and Alzheimer's disease. The increase in ROS is one of the reasons for the degeneration of dopaminergic neurons in the substantia nigra [96]. DEC1 has been identified as a retinoic acid-inducible gene in neuronal cells in recent years [8]. DEC1 expression is increased during oxidative damage. DEC1 overexpression is found to be sufficient to protect muscle cells from ROS-mediated death, up-regulate heme-oxygenase-1 (HO-1) expression, and down-regulate TNF-α expression, thereby protecting cells from oxidative damage [97]. Together, these results confirm that DEC1 can function as an antioxidant and confer protection to cells from ROS-mediated death, at least in part by regulation of HO-1 and TNF-α expression. Notably, the expression of DEC1 can be induced by PGE2 and retinoic acid in podocytes. Another study showed that DEC1 protects podocytes from oxidative stress by inhibition of the NADPH oxidoreductase enzyme [a major enzymatic source of ROS in podocytes [98] complex and induction of HO-1 [99]. These results suggest that DEC1 contributes to maintaining cell integrity against oxidative stress-mediated damage. PGC-1a is a transcriptional coactivator regulated by a variety of environmental stimuli, which functionally coordinates mitochondrial biogenesis and metabolic flux [100]. It has been found that HIF-dependent transcription inhibitor DEC1 inhibits PGC-1a-induced oxidative stress and ROS production. However, PGC-1a inhibition also interferes with mitochondrial respiration [101]. Therefore, in the pathogenesis of PD, whether DEC1 inhibits PGC-1α by antagonizing oxidative stress or inducing mitochondrial dysfunction remains to be further explored. In conclusion, DEC1 plays a beneficial role in combating ROS-mediated oxidative damage, and targeting DEC1 may have greater potential in combating oxidative stress in PD.

### 6.3 DEC1 Regulates Lipid Metabolism

It is now evident from genetic variant analysis that lipid and lipid transport pathways carry a significant and impactful genetic risk for age-dependent neurodegenerative diseases, such as PD and AD [102]. Relatively mild but persistent abnormalities in lipid levels in PD can lead to neurodegeneration later in life. Moreover, the physiological burden of elevated glucolipid levels in neurons may affect many organelles and pathways, including lipid membranes, vesicle transport, protein-protein interactions, autophagy clearance, and neuroinflammation [103]. Sterol regulatory element-binding protein 1(SREBP1) is an important transcriptional regulator of adipose synthesis genes. SREBP-1a and SREBP-1c are involved in the induction of lipogenic, fatty acid synthesis, and carbohydrate metabolizing genes. SREBP1 has been identified as a risk site for idiopathic PD in genome-wide association studies. And the loss of the SREBF1 pathway and deficient lipogenesis significantly reduce full-length PINK1 stabilization and inhibit mitophagy [104]. Studies have shown that DEC1 significantly inhibits the activity of the SREBP1 promoter, thereby reducing ATP-consuming anabolic lipogenesis [105]. There is evidence that active SREBP1a inhibits ABCA1 promoter activity, reduces macrophage ABCA1 expression and free cholesterol efflux, and blocks LXR-ligand-induced ABCA1 promoter activity [106]. Therefore, DEC1 may reduce lipid accumulation in macrophages by regulating the SREBP1/ABCA1 pathway. It has been reported that liver X receptors (LXRs), a nuclear receptor protein that forms heterodimers with retinoid X receptors (RXRs) [107, 108], have been reported to be a positive regulator of SREBP1c gene expression in liver and adipose tissue [109-111]. In addition, the apoptotic and neurotoxic effects of nonylphenol (NP) were accompanied by increased mRNA expression and protein levels of RXRα [112]. Importantly, DEC1 effectively suppresses activation of the RXR-LXR heterodimer and LXR target gene SREBP-1c and ABCA1 promoter activity [113]. Steroidogenic acute regulatory protein (StAR), a steroidogenesis-related gene, is known to be stimulated by HCG [114] and its transcription can be regulated by BMAL1 in steroidogenic tissues [115]. Overexpression of StAR significantly enhances macrophage cholesterol efflux to apolipoprotein AI (ApoA-I) by upregulating ABCA1 expression [116, 117]. Of note, the mRNA and protein levels of StAR are increased during hCG stimulation, and elevation of Dec1 gene expression is present at the same time [118]. These studies suggest that DEC1 is positively correlated with STAR. Additionally, CCAAT/enhancer-binding protein β (C/EBPβ) and peroxisome proliferator-activated receptor γ (PPARγ) are key transcription factors in adipogenesis and have been reported to be negatively regulated by DEC1 [119, 120]. Taken together, DEC1 serves as a crucial molecular regulator in the regulation of lipid metabolism and may be a potential therapeutic target for PD.

### 6.4 DEC1 Regulates the Immune System

The neuroinflammatory hallmarks of PD include reactive central nervous system myeloid cells, T cell infiltration into the CNS, and increased pro-inflammatory cytokines/chemokines in the blood, cerebrospinal fluid, or brain parenchyma of patients with PD [121]. A recent study found that some PD patients possess T cells that recognize specific epitopes derived from the PD-associated protein, α-synuclein (α-syn) [122]. And specific T cell reactivity to α-syn-derived epitopes is a feature of premotor and early motor PD [123]. Further study showed that knockout or pharmacological inhibition of T cells, specifically CD4 T cells, can reduce the major histocompatibility complex II protein expression on CNS myeloid cells and protect against TH^+^ neuron loss in the ipsilateral SNpc [121]. In recent years, relevant studies have found that DEC1 plays a critical role in regulating immune cell differentiation, controlling cytokine production, and maintaining the balance of pro- and anti-inflammatory signals due to its structural complexity and functional diversity [124]. DEC1 is an important factor in the maximal expression or inhibition of many early transcripts during T cell activation. Numerous studies have identified DEC1 as a key transcriptional mediator in the activation of naive CD4(+) T cells, which is required for the development of T cell-mediated autoimmune diseases. Interestingly, DEC1-deficient CD4+ T cells have cell-intrinsic defects in survival and proliferation. This phenomenon may be caused by direct or indirect effects of DEC1 transcriptional targets [19]. However, an initial study on DEC1-deficient mice showed defective T-cell-mediated recall responses and the development of spontaneous autoimmune disease caused by defects in activation-induced cell death [125]. These studies potentially suggest that DEC1 plays a different role in several stages of T cell activation, including clonal expansion, effector cytokine production, and activation cell clearance. Besides, in PD patients, the level of naturally occurring regulatory T (Treg) cells is reduced [126]. And the loss of Treg cells has been associated with rapid cognitive decline and disease progression in mouse models of neurodegenerative diseases such as Alzheimer's disease [127] and Amyotrophic lateral sclerosis [128, 129]. DEC1 combines with the transcription factor Runx1 to form a complex. Dec1/Runx1 complex binds regulatory elements in the IL-2Rα locus to up-regulate CD25 expression and increase the number of Treg cells [130]. This suggests that DEC1 is involved in the homeostasis of Treg cells and plays a role in inhibiting the development of spontaneous inflammatory responses. Taken together, inflammatory T cell responses appear to be more prevalent in the pre-symptomatic and early stages of PD. DEC1 may play a protective role in PD inflammatory response by regulating immune-related cells, but the specific mechanism of how DEC1 regulates T cells needs further study.

### 6.5 DEC1 Modulates Disorders of Glucose Metabolism

Some epidemiological studies have shown that diabetes is a risk factor for PD [131]. For example, dysglycemia can exacerbate motor symptoms in PD patients [132, 133]. The maintenance of normal blood glucose concentration mainly depends on the normal secretion of insulin and the normal coordinated activities of the CNS, endocrine glands, liver, gastrointestinal, and kidney. Abnormal blood glucose may be closely related to the cognitive impairment of non-motor symptoms in PD [134]. DEC1 is a transcription factor that inhibits gluconeogenesis by regulating phosphoenolpyruvate carboxykinase (PEPCK) gene expression [135]. Studies of rat liver cells have shown that insulin induces DEC1 transcription through the phosphoinositide 3-kinase (PI3-K) pathway [136]. As an important target gene of insulin, DEC1 is involved in insulin-mediated gene transcription regulation and human skeletal muscle inflammation *in vivo* [137]. ChREBP is a glucose-activated transcription factor involved in the regulation of fat production. Overexpression of DEC1 inhibits glucose and ChREBP-mediated upregulation of rat Fasn and liver pyruvate kinase (Lpk) mRNA [13]. In mice, fasting and refeeding modulate the DEC1-mediated circadian rhythms in peripheral tissues. These findings suggest that the expression of DEC1 is closely related to the metabolic activity of these tissues [138]. In rodent models of type 2 diabetes and insulin resistance, Liver X receptor (LXRs) activation normalizes blood glucose and improves insulin sensitivity, mainly due to the inhibition of hepatic gluconeogenesis [139]. Meanwhile, ligand-dependent-LXR binds to the promoter of DEC1 and regulates the expression of the liver clock system and metabolic genes [82]. In summary, DEC1 is a major physiological regulator of metabolic and energy balance. DEC1 regulates dysglycemia and maintains normal blood glucose levels, and it may be a potential target for regulating glucose metabolism disorders in PD.

## CONCLUSION

Parkinson’s disease is a heterogeneous disease with rapidly and slowly progressive forms. Currently, there is neither a clinical biomarker for early detection, diagnosis, or prognosis, nor a cure or disease‐modifying treatment available for PD. Current treatments are more symptomatic, involving pharmacologic approaches (typically with levodopa preparations prescribed) and nonpharmacologic approaches (such as exercise and physical, occupational, and speech therapies). Deep brain stimulation, levodopa-carbidopa enteral suspension, Stem cell transplantation, and other surgical approaches are typically considered when individuals with Parkinson’s disease experience do not respond to medication adjustments [3, 140]. Biomarkers and disease-modifying therapies are both urgent unmet medical needs in the treatment of PD. The one-gene-one-target approach helps identify new biomarkers that can track or predict disease progression [141]. This review provides ample evidence that DEC1 may play a protective role in multiple aspects of PD pathogenesis or progression by regulating its target gene expression. These target genes of DEC1 are involved in the modulation of cell apoptosis, oxidative stress, lipid metabolism, immune balance, and glucose metabolism.

DEC1, a member of the basic helix–loop–helix (bHLH) superfamily, has attracted growing attention since it was discovered in 1997. Although most studies have demonstrated a beneficial role of DEC1 in the nervous system, there are also studies showing paradoxical roles in other systems. For example, DEC1 is widely used as a marker of senescence *in vivo* and can directly induce senescence in cultured cells [142, 143]. However, age is also a risk factor for PD. In tumors, expression of DEC1 is thought to be induced by hypoxia and is up-regulated in several kinds of malignancies, including breast [144], stomach [145], lung [146], liver [147], and colon cancer [88]. More importantly, DEC1 expression is increased in oligodendroglia neoplasms [148] and glioma cells [149]. Also, attenuation of DEC1 inhibits the malignant growth of glioma. So, the close relationship between DEC1 and tumors is detrimental to PD. Another study found that DEC1 is a negative regulator of B cell activation and development [150]. It's worth noting that a recent study found that proliferating B cell counts are decreased in patients with PD compared with controls, whereas proportions of proinflammatory cytokine-producing B cells are increased [151]. This result reflects that DEC1 negatively regulates B cell development and is detrimental to PD. In conclusion, the relationship between DEC1 and PD is somewhat complicated. The pleiotropic and puzzling effects of DEC1 in PD may be attributed to several reasons. First, DEC1 may switch between activator and repressor functions in transcriptional regulation in response to different stimuli. Second, DEC1 expression is altered by genomic instability because the DEC1 gene is located at 3p26, a hot spot for chromosomal mutations in many tumors. Third, DEC1 can regulate the expression of multiple target genes on one side, and the expression of DEC1 on the other side can also be induced by a variety of stimuli, such as multiple transcription factors, growth factors/hormones, and cytokines, as well as environmental stimuli, including hypoxia, light pulse, and nutrients, which underlies the complex effects of DEC1. Fourth, DEC1 activity is not only regulated by its protein level but also by posttranslational modifications, such as SUMOylation and phosphorylation, suggesting that the modulation of DEC1 itself is complicated. Fifth, PD progresses in several stages, and its pathogenesis is extremely complex, especially when it intersects with the immune system and metabolic system. Nevertheless, increasing evidence supports the view that DEC1 may serve as a protective factor against PD. It is hoped that the potential role of DEC1 protein in PD will provide further targets for the development of new therapeutic candidates and biomarkers.

## FUTURE PERSPECTIVES

DEC1 is expressed in many tissues and cell types of various animals, including the midbrain, cerebellum, hypothalamus, cerebral cortex, macrophages, neurons, and astrocytes, …among other and have multiple effects on PD. The activated PI3K/Akt signaling fosters endothelial survival, limits neuronal injury, and blocks inflammatory neuron death during the pathophysiological process of PD. DEC1 reciprocally interacts with PI3K/Akt signaling pathway to inhibit neuronal apoptosis, resulting in neuroprotective effects. This is achieved because DEC1, Sp1, and PI3K/Akt may form a functional complex leading to transactivation. It remains to be determined whether DEC1 is bound to the specific sequence in promoters of PI3Kp110α and its downstream targets. Given its anti-PD properties, increasing the level of DEC1 in the brain may have new implications for the treatment of PD. But it is not feasible to convert the recombinant DEC1 protein into an active drug. In addition, there are currently no synthetic activators or agonists specific to DEC1 in animal models of PD. Therefore, strategies aimed at stimulating endogenous DEC1 production may be more valuable. A recent study has found that miR-141-3p is up-regulated in PD cell models induced by 1-methyl-4-phenylpyridinium- (MPP+-) [152], indicating that miR-141-3p might involve in the progression of PD. Importantly, bioinformatics analysis identified the possible binding of the up-regulated miR-141-3p to the DEC1 3’UTR. miR-141-3p has been identified as a regulatory target of the DEC1 gene [153]. Thus, *in vivo* delivery of miR-141-3p inhibitors upregulate DEC1 expression and may have potential therapeutic benefits in the treatment of PD.

In the future, the exact role of DEC1 in PD needs to be further studied. Also, more work is needed to elucidate how to most effectively target DEC1, either *via* regulation of expression or *via* posttranslational modification. Although studies have reported the beneficial effects of enhanced DEC1 expression, it is difficult to predict the most effective method to promote the protective effect of DEC1 in PD. In addition, there are several debatable and critical questions that need to be answered: (1) What and how to regulate DEC1 expression during the occurrence and development of PD? (2) Are there any other DEC1 target genes that can directly affect the development of PD? (3) Is DEC1 involved in the inflammatory response in PD progression? (4) Since age is an important factor affecting the function of DEC1, whether an increase in DEC1 can aggravate the deterioration process of neuronal cells due to the impairment of neural repair function caused by aging? Finally, there are few studies on DEC1 in the brain, so it is very important to strengthen the research on DEC1 in the CNS. The answers to these questions will certainly provide meaningful insights into the roles of DEC1 in PD and make DEC1 an attractive target for the treatment of PD.

## LIST OF ABBREVIATIONS

BDNF: Brain-derived Neurotrophic Factor
CNS: Central Nervous System
HO-1: Heme-oxygenase-1
PD: Parkinson's Disease
SNpc: Substantia Nigra Pars Compacta
TrkB: Tyrosine Kinase B
